# Sex differences in three-dimensional intra-cycle velocity fluctuation and performance during freestyle swimming among high-level swimmers

**DOI:** 10.1038/s41598-026-48979-1

**Published:** 2026-04-29

**Authors:** Zhenyu Jin, Yulin Zhou, Yuhang Zhou, Qian Yu, Dapeng Wang, Sijia Shen, Yuhong Wen

**Affiliations:** 1 School of Sports, Leisure and Tourism, Beijing Sport University, 48 XinxiRoad, Haidian District, Beijing, 100084 China; 2 Key laboratory of Sport Training of General Administration of Sport of China, Beijing, 100084 China; 3 Department of Public Physical and Art Education, Zhejiang University, Hangzhou, 310030 Zhejiang China; 4 College of Sports Science, Beijing Sport University, Beijing, 100084 China; 5 College of Education, Beijing Sport University, Beijing, 100084 China; 6 Physical Education and Research Department, Peking University, Beijing, 100871 China

**Keywords:** High-level swimmers, Freestyle, Intra-cycle velocity fluctuation, Sex differences, 3D motion capture, Health care, Physiology

## Abstract

This study examined sex differences in three-dimensional intra-cycle velocity fluctuations (IVFs) of the body’s center of mass and the relationship between IVFs and swimming velocity (Vswim) in high-level swimmers during freestyle. Maximal-effort freestyle sprints performed by 13 high-level athletes (7 males and 6 females) were captured using a Qualisys three-dimensional motion capture system. Given the limited sample size, robust statistical approaches, including Welch’s *t*-tests, Bayesian analyses, and permutation tests were applied. Male swimmers exhibited significantly higher Vswim (*p* < 0.001, ES = 2.94), forward IVF (*p* < 0.001, ES = 2.47), and vertical IVF (*p* = 0.022, ES = 1.57) compared with female swimmers. Bayesian analysis of interaction terms revealed no statistically significant moderating effect of sex on the relationship between IVF and Vswim. These findings indicate that although clear sex differences are evident in specific IVF components and swimming speed among high-level swimmers, the functional relationship between IVF and performance remains statistically consistent across sexes. From a practical perspective, IVF-based interventions should integrate sex-related physiological characteristics with individualized technical profiling to avoid overgeneralization. This study also establishes a high-fidelity methodological reference for three-dimensional kinematic analysis, providing a valuable dataset for future research and technological validation.

**Supplementary Information:**

The online version contains supplementary material available at 10.1038/s41598-026-48979-1.

## Introduction

Swimming performance is commonly quantified by time or speed, both influenced by technical execution and physical conditioning^1,2^. Higher-level swimmers exhibit greater ability to control velocity stability beyond achieving higher speeds^3–5^. Speed regulation involves macro-level coordination of stroke rate and length^2,6–8^ and micro-level control of intra-cycle velocity fluctuation (IVF)—a mechanically meaningful dimension of propulsive stability^9^. As a key parameter linking speed with stroke technique and energy expenditure^10,11^, IVF is sensitive to performance level and indicates technical proficiency^12,13^.

The precise relationship between IVF and performance remains debated^11,14,15^. Interpreting such complex biomechanical relationships requires rigorous statistical frameworks to distinguish signal from noise^16^. Some studies associate lower IVF with superior performance^13,17–20^, while others report no significant association^21,22^ or even higher IVF among better-performing swimmers^23^. These discrepancies stem from variations in data acquisition systems, velocity measurement locations (e.g., hip vs. center of mass [CoM]), analytical techniques, pacing strategies, and fatigue states^18,24–26^. The sensitivity of IVF to measurement precision is reflected in reported values ranging from over 25% to below 10%^17,27^. Notably, most existing research has focused on male swimmers, with a scarcity of sex-comparative studies^11^.

A major methodological challenge is accurately capturing three-dimensional whole-body kinematics in water. Although some studies have used marker-based methods, they relied on hip velocity data^19^, which may not reflect CoM motion. High-precision 3D motion capture is the gold standard in terrestrial biomechanics but remains underutilized in swimming due to technical constraints^28^. Moreover, research has largely focused on forward IVF, overlooking its three-dimensional nature and the distinct influences of different motion planes on performance^11^.

Sex may systematically affect both the magnitude of IVF and its functional association with performance due to physiological differences in muscle composition, body morphology, and metabolic profiles^10,11,29^. However, empirical evidence on sex-specific IVF patterns remains limited. To address this, we employed high-precision 3D motion capture to develop a full‑body kinematic model and derive CoM velocity during freestyle. The aims are to (1) establish a high‑fidelity CoM kinematics dataset; (2) examine sex differences in IVF and performance; and (3) test whether sex moderates the IVF‑performance relationship. This work also proposes a standardized framework for capturing whole‑body CoM kinematics, providing a validated reference for future studies.

We hypothesized that (1) significant sex differences exist in performance and three-dimensional IVF, and (2) sex moderates the IVF‑performance relationship. This study aims to clarify the debated role of IVF in elite swimming and provide a biomechanically robust foundation for sex‑inclusive technical training.

## Methods

### Participants

Thirteen high-level swimmers (7 males, 6 females) with a mean age of 22.4 ± 2.8 years participated in the study. Anthropometric and performance characteristics are summarized as follows: male participants had an average height of 1.83 ± 0.03 m and a weight of 77.84 ± 5.31 kg, while female participants averaged 1.73 ± 0.05 m in height and 64.43 ± 4.65 kg in weight. Regarding competitive level, male swimmers’ World Aquatics points for their primary event averaged 746.08 ± 58.33, with personal bests in the 50-m freestyle (long course) corresponding to 87.42 ± 2.56% of the world record. Female swimmers had an average World Aquatics points score of 770.12 ± 46.89 for their primary event, with their best 50-m freestyle performances equating to 87.12 ± 2.35% of the world record.

All participants had extensive experience in systematic swimming training. During the testing period, they were not undergoing high-intensity training cycles but maintained a weekly minimum of five weekly sessions, totaling at least 20,000 m per week. Before testing, all participants were confirmed to be free of injuries and illnesses. Written informed consent was obtained from each participant. The study protocol was approved by the Ethics Committee for Sports Science at Beijing Sport University (Approval No. 2023174 H) and conducted in accordance with the Declaration of Helsinki and relevant ethical guidelines.

### Testing procedure

Testing was conducted in a standard 50-m indoor pool. To optimize data acquisition, lane lines were removed from the designated testing area. Before formal data collection, each participant completed an individualized warm-up routine, comprising both onshore and in-water components designed to simulate competition preparation. The warm-up was supervised by researchers to ensure participants achieved sufficient physiological and technical readiness.

Following the warm-up, participants’ skin was dried, and reflective markers were attached according to a full-body marker set. Brief onshore movements were performed to allow participants to adapt to the markers. Additional markers were strategically placed to improve modeling accuracy during dynamic swimming.

For formal testing, participants first assumed a static underwater posture to capture a reference skeletal model. They then swam slowly to a position approximately 35 m from the starting end of the pool. From this position, participants performed a maximal-effort freestyle sprint toward the starting end, with motion data collected within the calibrated capture volume 5–15 m from the start.

### Data acquisition

Motion data were captured using a Qualisys motion capture system (Qualisys AB, Gothenburg, Sweden), consisting of 10 underwater cameras (Oqus 700+, 12 mm lens) and 13 surface cameras (Aqus 12, 8 mm lens), sampling at 100 Hz, which meets standard criteria for kinematic data acquisition^30,31^. Owing to the multi-day testing schedule, the system was spatially recalibrated at the start of each testing session, with calibration errors consistently maintained below 3 mm, which aligns with recommended protocols for minimizing measurement error in kinematic systems^32^. The reliability of the equipment and procedures has been validated in previous studies^19,33^.

**Fig. 1:**
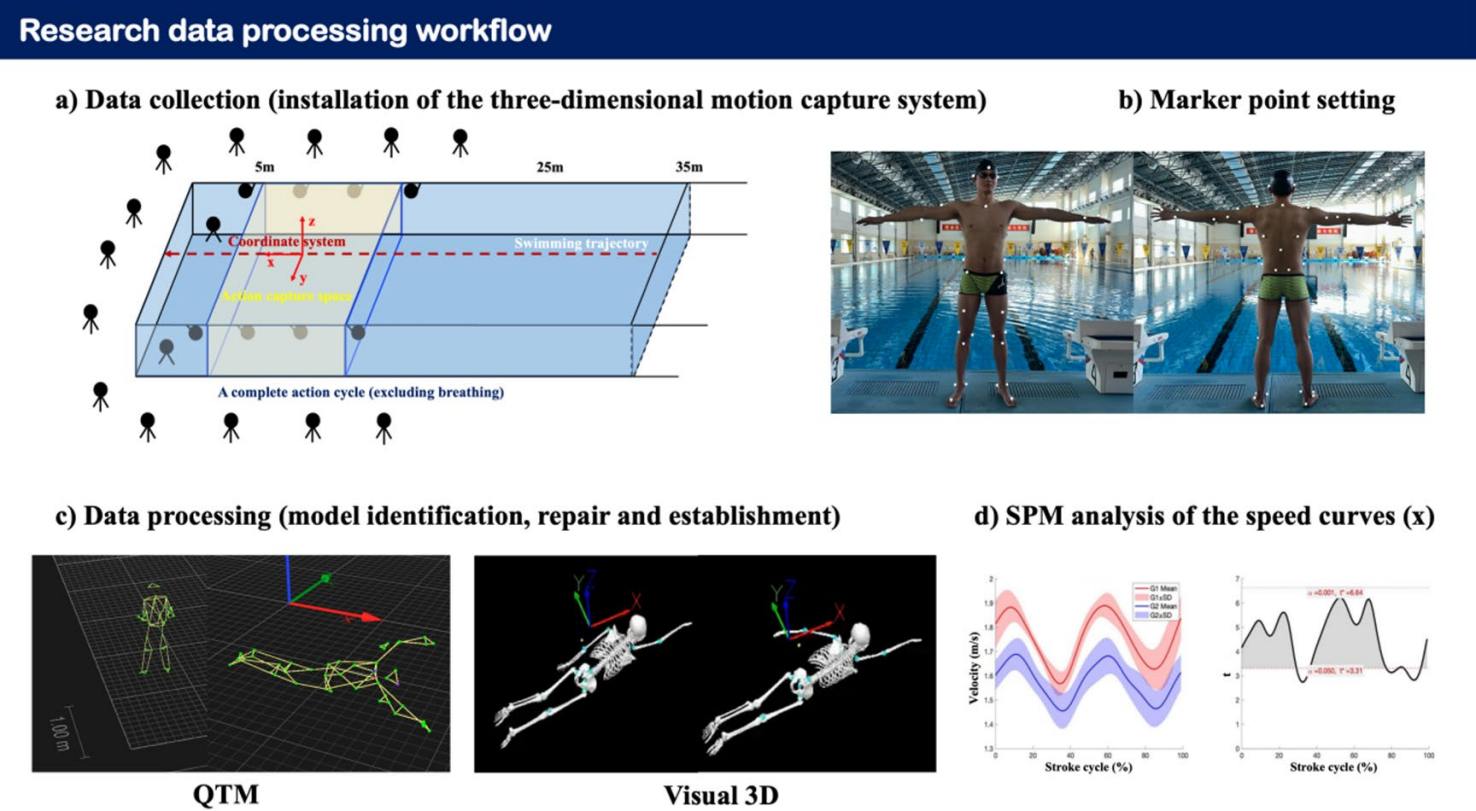
Research data processing workflow.

The coordinate system was defined as follows: the x-axis aligned with the swimming direction (toward the starting end), the y-axis represented the lateral direction, and the z-axis indicated the vertical direction Fig. 1(a). Full-body reflective marker placement is illustrated in Fig. 1(b), based on the Qualisys full-body marker set guidelines (Supplementary Material), with additional markers on the upper arms to optimize tracking during swimming^33^.

The calibrated capture volume, verified using Qualisys Track Manager software, extended from 5 to 15 m from the starting end. To ensure high precision in kinematic analysis, only data within this calibrated volume were used for subsequent calculations. This segment was selected because it corresponded to the phase after participants had completed their initial acceleration and reached a stable maximal swimming velocity.

### Data processing

Reflective marker trajectories from both static calibration and dynamic swimming trials were processed using Qualisys Track Manager software. Aquatic motion capture presents inherent challenges, including frequent marker occlusion, water-surface reflections, and the dynamic nature of swimming, resulting in substantial gaps and labeling errors in the initial automatic tracking output. To maximize accuracy, all marker trajectories were manually identified, inspected, and corrected on a frame-by-frame basis. After trajectory refinement, a full-body skeletal model was constructed in Visual3D (version 2023.01.0, C-Motion, Inc., Germantown, MD, USA), which has been validated for aquatic applications^19,33^. Raw marker trajectories were filtered using a fourth-order Butterworth low-pass filter with a cutoff frequency of 6 Hz^34^. The complete workflow for marker tracking, model construction, and data interpolation is summarized in Fig. 1.

Due to marker occlusion from limb-to-body contact, full-body model reconstruction across consecutive cycles was not feasible; thus intra-session reliability was not assessed.

### Data calculation

The CoM position was estimated using Zatsiorsky-Seluyanov human body inertia parameters. Mean swimming velocity (Vswim) and IVF were then calculated from the CoM kinematics. A complete freestyle stroke cycle was defined as the interval between two consecutive entries of the same-side hand. Instantaneous velocity data within this cycle were extracted for analysis, with none of the selected cycles including breathing. This approach is widely used and considered representative in the field^19,35,36^.

This study mainly used traditional dispersion measures to assess IVF. Dispersion metrics are widely employed in kinematic research due to their simplicity and interpretability. Given growing concerns about the use of the coefficient of variation for assessing IVF^14,15^, the standard deviation of instantaneous velocities within the stroke cycle^19^ was selected as the IVF metric for each directional component (x, y, z), minimizing the potential influence of mean velocity variations. This approach aligns with recent methodological developments in swimming biomechanics, where accelerometry has been employed to detect fatigue-induced variations in technique among high-performance athletes^37^. By integrating this reference, we acknowledge the value of complementary measurement techniques while retaining a focus on traditional kinematic descriptors relevant to the present research questions. Additionally, to align with the growing use of statistical parametric mapping (SPM) in movement analysis, a supplementary SPM analysis was conducted—limited to forward-direction (x-axis) IVF time-series data—and is presented in the methodological figures for comparative illustration (Fig. 1d)^38,39^.

### Statistical analysis

Statistical analyses were conducted in R (version 4.2.2) and RStudio (2023.03.0 + 386) using robust methods suitable for the small sample size (*N* = 13; 7 males, 6 females) without assuming normality or homoscedasticity. Analyses were performed in three phases.

Phase 1: Sex Differences. Welch’s independent samples *t*-tests were used to compare males and females on mean swimming velocity (Vswim) and directional IVFs (x, y, z). Effect sizes were calculated using Cohen’s *d*. Complementary Bayesian independent samples *t*-tests provided Bayes factors (BF_10_) to quantify evidence for or against the null hypothesis of no difference.

Phase 2: IVF-Performance Relationship Visualization and Slope Comparison. Associations between each directional IVF component and Vswim were visualized using sex-stratified scatter plots. Sex-specific linear regression slopes were estimated via bootstrap resampling (5,000 replicates) with 95% percentile confidence intervals. Differences between male and female slopes for each IVF direction were assessed using a permutation test (10,000 iterations) under the null hypothesis of equal slopes.

Phase 3: Testing Sex as a Moderator. Bayesian robust linear regression (using the brms package) was employed to assess whether sex moderated the relationship between each IVF component and Vswim. Models included a sex × IVF interaction term and used Student-*t* error distributions to account for potential outliers. Weakly informative priors were specified: Normal(0, 1) for regression coefficients (including the interaction *β*) and Exponential(1) for the scale (*σ*) and degrees-of-freedom (*ν*) parameters. These priors were selected to constrain parameter estimates to plausible ranges without overly influencing the posterior distributions, which is appropriate given the exploratory nature of the study and the small sample size^40^. Four Markov chains were run (4,000 iterations each, with 1,000 warm-up iterations), and convergence was confirmed (R̂ < 1.01). Sensitivity analyses were conducted to evaluate robustness to alternative priors (e.g., Normal(0, 2), Student-*t*(3, 0, 1)). Evidence for moderation was assessed using two criteria: (1) Bayes factors (BF_10_) comparing models with and without the interaction term, and (2) whether the 95% highest density credible interval (HDI) for *β*_interaction_ excluded zero.

### Statistical size considerations for future research

Given the exploratory nature of this study and the lack of established effect size estimates in the literature, a prospective sample size calculation was not performed. To provide guidance for future research, the sample size required per group to achieve 80% power for each observed effect size was calculated based on the observed effect sizes and sample sizes. Analyses were conducted using G*Power 3.1.9.7 (“difference between two independent means,” allocation ratio = 6/7). Results are reported alongside the primary findings in the section “Sex differences in IVF and performance”.

## Results

### Sex differences in IVF and performance

Descriptive statistics for swimming velocity (Vswim) and three-directional IVFs (IVFx, IVFy, IVFz) in male (*n* = 7) and female (*n* = 6) high-level swimmers are presented in Table 1. Male swimmers showed higher mean values than females for Vswim (1.75 ± 0.04 m/s vs. 1.58 ± 0.06 m/s), IVFx (0.12 ± 0.02 m/s vs. 0.08 ± 0.01 m/s), and IVFz (0.10 ± 0.02 m/s vs. 0.07 ± 0.02 m/s). In contrast, mean IVFy was similar between groups (0.07 ± 0.02 m/s for both).

Welch’s *t*-tests indicated significant sex differences for Vswim (*t* = 5.10, *p* < 0.001), IVFx (*t* = 4.67, *p* < 0.001), and IVFz (*t* = 2.76, *p* = 0.022), but not for IVFy (*t* = −0.21, *p* = 0.835). Corresponding effect sizes were large for Vswim (Cohen’s *d* = 2.94), IVFx (*d* = 2.47), and IVFz (*d* = 1.57), and negligible for IVFy (*d* = −0.12). These findings are summarized visually in Table 1.

**Table 1:** Descriptive Statistics of Swimming Velocity (Vswim) and Intra-cycle Velocity Fluctuations (IVF) in Male and Female High-Level Swimmers.

Variable	Group (*n*)	Mean ± SD	Min	Max	Welch’s t	*p*	Cohen’s d
Vswim	Male (7)	1.75 ± 0.04	1.69	1.80	5.10	< 0.001	2.94 [1.97, 6.15]
	Female (6)	1.58 ± 0.06	1.49	1.68			
IVFx	Male (7)	0.12 ± 0.02	0.10	0.15	4.67	< 0.001	2.47 [1.91, 4.41]
	Female (6)	0.08 ± 0.01	0.07	0.09			
IVFy	Male (7)	0.07 ± 0.02	0.04	0.11	−0.21	0.835	−0.12 [−1.57, 1.01]
	Female (6)	0.07 ± 0.02	0.03	0.10			
IVFz	Male (7)	0.10 ± 0.02	0.07	0.12	2.76	0.022	1.57 [0.40, 5.77]
	Female (6)	0.07 ± 0.02	0.05	0.11			

*SD* standard deviation, *IVFx, y, z* intra-cycle velocity fluctuation in the forward, lateral, and vertical directions, respectively.

Bayesian *t*-tests provided strong corroborative evidence: extremely strong support for sex differences in Vswim (BF_10_ = 82.41) and IVFx (BF_10_ = 28.48), substantial evidence for IVFz (BF_10_ = 3.71), and support for the null hypothesis of no difference in IVFy (BF_10_ = 0.46).

To inform future study planning, the sample sizes required to achieve 80% power for detecting the observed effect sizes are presented in Table 2. For the large effect sizes observed in Vswim and IVFx, approximately 12 participants per group would be sufficient, whereas the effect size observed for IVFz (d = 1.57) would require approximately 24 participants per group under similar experimental conditions. As expected, the negligible effect observed for IVFy would require an impractically large sample (approximately 3,634 per group), indicating that meaningful sex differences in lateral IVF are unlikely to exist in this population.

**Table 2:** Sample Size Requirements per Group to Achieve 80% Power for Detecting Observed Sex Differences.

Variable	Observed Cohen’s d	Post-hoc Power (1-β)	Required *N* per group for 80% Power*
Vswim	2.94	0.98	12
IVFx	2.47	0.97	12
IVFy	−0.12	0.06	3634
IVFz	1.57	0.95	24

*Calculations based on two-tailed independent samples *t*-tests (Welch’s procedure) with *α* = 0.05, power = 0.80, observed effect sizes, and an allocation ratio of N_2_/N_1_ = 6/7. Post-hoc power values are reported for completeness but should be interpreted with caution due to their inherent circularity (they are directly derived from the observed *p*-values). The required sample size per group is the primary guidance for designing future studies.

### Influence of sex on the IVF-performance relationship

**Fig. 2:**
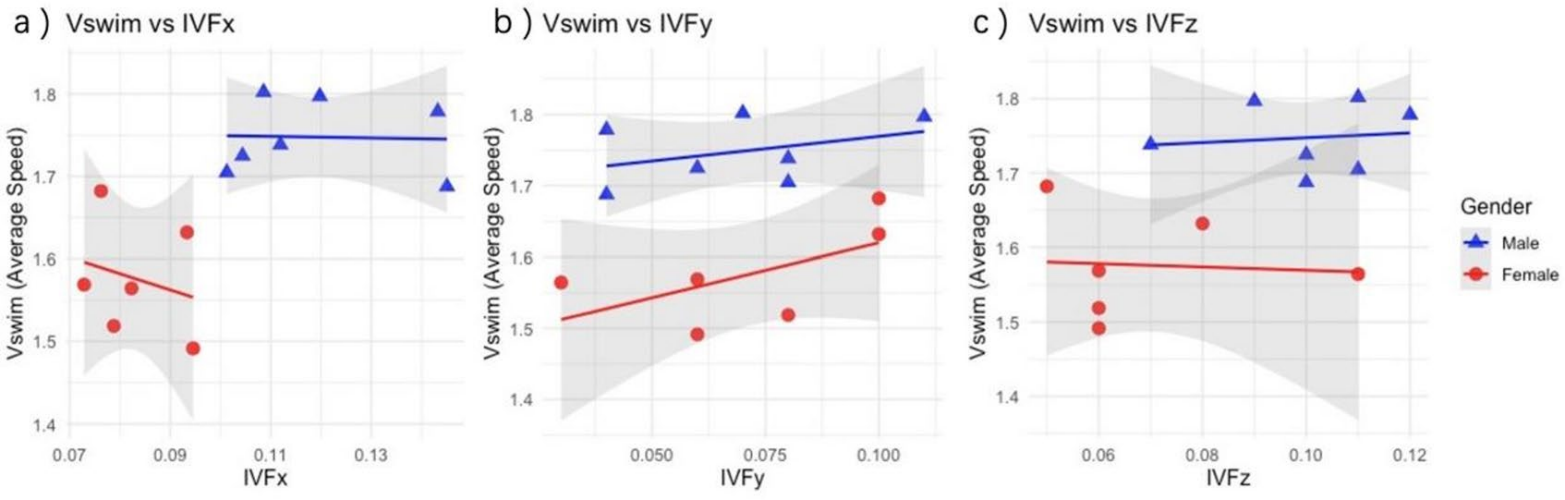
Scatter plots showing the relationship between directional IVF (x, y, z) and swimming velocity (Vswim) in male and female swimmers.

To evaluate whether sex moderates the relationship between IVF and swimming performance (Vswim), we first visualized these associations using scatter plots with sex-specific regression lines (Fig. 2). The plots suggested possible differences in slope magnitude between males and females, particularly for IVFx and IVFz.

Permutation analysis revealed no statistically significant sex differences in regression slopes for any direction: IVFx (Δ*β* = 1.86, *p* = 0.246), IVFy (Δ*β* = − 0.84, *p* = 0.624), and IVFz (Δ*β* = 0.55, *p* = 0.409). These findings indicate that, despite observed sex differences in absolute IVF values (Section “Sex differences in IVF and performance”), the functional relationships between IVF and performance are consistent across sexes. Notably, although visual inspection of Fig. 2 suggested that male and female swimmers exhibited opposite slope directions for the IVFx-Vswim and IVFz-Vswim relationships, formal analyses did not detect statistically significant moderation by sex. This discrepancy between graphical patterns and inferential results highlights the need for further investigation with larger samples to determine whether subtle, sex-specific associations exist in these directional components of IVF.

Bayesian robust regression provided additional evidence against a moderating effect of sex. The 95% HDIs for interaction terms included zero: IVFx [–2.08, 1.75], IVFy [–0.98, 1.91], and IVFz [–1.74, 1.49]. Corresponding Bayes factors offered anecdotal support for the null model (no interaction): IVFx (BF_10_ = 0.96), IVFy (BF_10_ = 0.94), and IVFz (BF_10_ = 0.849). Together, these results indicate a lack of reliable evidence for sex moderation of the IVF-performance relationship in high-level freestyle swimmers. Sensitivity analyses confirmed that these findings were robust across alternative prior specifications, with all interaction term 95% HDIs remaining inclusive of zero.

## Discussion

### Sex differences in three-dimensional intra-cycle velocity fluctuations

This study revealed significant sex differences in freestyle swimming performance and specific components of IVF among high-level swimmers. Male swimmers demonstrated faster mean swimming velocity (Vswim) and greater forward (IVFx) and vertical (IVFz) velocity fluctuations compared with females, whereas lateral fluctuation (IVFy) showed no significant difference. These findings suggest that, within this elite cohort, higher performance in short-distance freestyle is associated with a more pronounced intra-cycle velocity pattern, particularly in the forward and vertical directions.

The higher IVFx in males aligns with some reports on sprint performance^23^, but direct evidence linking forward IVF to superior performance remains inconsistent: some studies report negative associations^20^, while others find no relationship^21,23^. These discrepancies likely reflect methodological differences in measurement points (hip vs. CoM) and analytical protocols^18,24^. The present study addresses these by using full-body CoM kinematics from high-precision 3D motion capture, providing a more accurate representation, as hip-based estimates overestimate vertical displacement compared to CoM^41^.

The greater IVFz observed in males may be biomechanically attributed to sex-related differences in lower-limb power and kick amplitude^42^, as well as the magnitude of body roll, which influences vertical trunk displacement. Although direct evidence linking these specific factors to IVFz magnitude is limited, the observed absence of sex moderation in the IVFz-performance relationship suggests that minimizing non-propulsive vertical oscillations remains a universal technical objective for maximizing speed, a notion supported by the negative correlation between IVFz and swimming velocity^43^.

Physiologically, these sex differences in IVF likely reflect variations in force production capacity, body composition, and metabolic profiles. Greater muscle mass and a higher proportion of type II fibers in males^44^ may enable more powerful propulsive phases^45^, increasing IVFx and IVFz. Females’ generally higher body fat percentage and distinct morphology may influence swimming performance through multiple pathways. These characteristics not only potentially enhance buoyancy and passive hydrodynamic efficiency but also interact with factors that are known to directly affect the IVF-performance relationship, such as energy cost and propelling efficiency^10,46–48^.

Methodologically, this study builds on previous work by quantifying three-dimensional CoM kinematics rather than relying on hip-point tracking. While hip kinematics have been validated for overall stroke analysis^35^, they may overestimate vertical displacement^41^, necessitating whole-body CoM assessment for accurate IVFz quantification. The resulting high-fidelity dataset provides a validated reference for future studies employing more accessible measurement tools.

In summary, although sex differences in IVF magnitude are evident among elite freestyle swimmers, they likely reflect underlying physiological and technical adaptations rather than fundamentally distinct biomechanical principles. The strong association between IVFx and performance in males—and its sensitivity to methodological choices–highlights the importance of standardized, CoM-based assessments in future sex-comparative research.

### Absence of sex moderation in the IVF-performance relationship

A key finding of this study is the absence of a moderating effect of sex on the relationship between IVF and swimming performance (Vswim). Although significant sex differences were observed in the absolute magnitudes of Vswim, IVFx, and IVFz (Section “Sex differences in three-dimensional intra-cycle velocity fluctuations”), both frequentist (permutation tests) and Bayesian robust regression analyses showed that the slopes linking each directional IVF component to Vswim did not differ significantly between males and females. In the Bayesian analyses, the 95% HDIs for all sex × IVF interaction terms included zero, and Bayes factors provided support for the null models (no interaction).

This suggests that the fundamental biomechanical relationship between velocity fluctuation and mean swimming speed is preserved across sexes. The contrast between group differences in absolute values and the consistency of functional relationships indicates that male and female swimmers share a common performance continuum, where IVF acts as a technical descriptor whose association with speed is not inherently sex‑specific.

The absence of observed moderation can be understood from several complementary perspectives. First, it reflects the high degree of technical individualization characteristic of elite swimmers. Elite performance is shaped by highly individualized movement patterns^49^, supporting the need for personalized analysis. IVF arises from each swimmer’s unique balance of propulsion, drag, and coordination^11,39^. Even within elite cohorts, considerable inter-individual variability exists, making a universal “optimal” IVF unlikely. Performance can be optimized either by minimizing fluctuations for mechanical efficiency or by strategically exploiting larger fluctuations for propulsive impulse, depending on the swimmer’s force production and coordination^20,50^. This principle of individualized optimization appears to operate similarly in both sexes.

Second, the high methodological precision afforded by full-body CoM kinematics used in this study may provide a more reliable foundation for identifying common biomechanical principles. Previous inconsistencies in the IVF-performance relationship may partly reflect methodological noise, such as relying on hip-point proxies that poorly approximate CoM motion, particularly in the vertical axis^35^. By establishing a high-fidelity kinematic reference, the present study reduces such noise, potentially uncovering the underlying consistency across sexes.

Statistical equivalence in the IVF-performance slope does not mean that physiological sex differences are irrelevant for training. On the contrary, well-established distinctions in muscle composition, strength, body morphology, and metabolic profiles^29^ should guide individualized training. For example, male swimmers may benefit from interventions that enhance power during propulsive phases, whereas female swimmers may gain more from technical refinements that improve efficiency and stability. The key takeaway is that coaches should tailor training by integrating each athlete’s physiological profile with their individual IVF characteristics, rather than relying on broad sex-based generalizations, a strategy consistent with recent performance monitoring frameworks in competitive swimming^51^.

Therefore, the absence of a statistically significant moderating effect should not be interpreted as justification for sex-generalized training prescriptions. This null finding may reflect the limited sample size, high inter-individual variability among high-level swimmers, and reduced statistical power to detect interaction effects. As a result, training should remain athlete-centered, incorporating individualized 3D IVF profiles alongside each swimmer’s specific physiological and technical characteristics, rather than relying on broad sex-based strategies. Future research with larger cohorts is needed to investigate potential subtle moderating effects and to establish optimal IVF ranges within a sex-sensitive framework.

### Methodological contribution and reference role

This study has inherent limitations that also inform future research. The small elite sample restricts generalizability and reduce the ability to detect subtle effects, underscoring the need for replication in larger cohorts.

Although this study provides detailed CoM kinematics, the absence of concurrent physiological or force measurements limits mechanistic interpretation. Future research should combine high-fidelity kinematics with kinetic and physiological data to achieve a more comprehensive understanding.

Although the analysis was three-dimensional, it relied on discrete IVF metrics. Continuous approaches, such as SPM, could provide deeper insights into coordination dynamics throughout the stroke cycle.

Logistical constraints prevented assessment of test-retest reliability; future studies should include this where feasible.

Overall, future research should prioritize larger sample sizes, integration of multimodal data, application of this study’s reference model for more detailed analysis, and the translation of laboratory findings into practical training tools.

### Limitations and future directions

First, the small sample size limits the precision of estimates and generalizability, a common challenge in research involving elite athletes. To address this, robust statistical methods (e.g., Welch’s *t*-tests, Bayesian analysis, and permutation tests) were employed, specifically chosen for small-sample inference, thereby strengthening the internal validity of the comparisons. Although statistical power was high for the observed large effects, replication in larger cohorts is needed to confirm and extend these findings.

Second, while detailed CoM kinematics were captured, the absence of concurrent measurements of physical capacities or propulsive forces restricts a fully mechanistic interpretation of the observed sex differences. Nonetheless, the high-fidelity, full-body 3D kinematic dataset established in this study provides a critical foundation for future research integrating physiological and kinetic measures.

Third, although the analysis captured three-dimensional velocity fluctuations, it relied on discrete IVF metrics. Extending these analyses to continuous profiles across the entire stroke cycle, using approaches such as SPM, could provide deeper insights into inter-segment coordination^14,38,39^.

Finally, test-retest reliability was not assessed due to the extensive time required for manual trajectory reconstruction and frequent marker occlusion from limb-to-body contact. Methodological frameworks for such reliability are available^32,52^, but intra-session ICC could not be implemented here. Future studies should address this.

Future research should prioritize larger cohorts to identify optimal IVF ranges and integrate time-frequency analyses with biomechanical modeling, supporting personalized, data-driven training frameworks.

## Conclusion

Using 3D motion capture, this study investigated sex differences in three-dimensional IVF and swimming performance in high-level freestyle swimmers. Males exhibited higher swimming velocity, forward IVF, and vertical IVF than females, whereas lateral IVF showed no difference. Sex did not moderate the IVF–performance relationship, indicating a similar biomechanical link across sexes, though this does not justify sex‑based training generalizations. Integrating individualized IVF profiles with performance monitoring frameworks supports athlete‑centered technical development. This study also provides a standardized, high‑fidelity kinematic dataset as a methodological reference for future research.

## Supplementary Information

Below is the link to the electronic supplementary material.

Supplementary Material 1

## Data Availability

The data can be obtained by contacting the first author or the corresponding author of the article.
