# Supplementary material for: Sex differences in three-dimensional intra-cycle velocity fluctuation and performance during freestyle swimming among high-level swimmers
Source: Sci Rep. 2026 Apr 29;16:20027. doi: 10.1038/s41598-026-48979-1 (PMC13319195; doi:10.1038/s41598-026-48979-1)
Supplement: Supplementary file 1 — Supplementary Material 1 [file 41598_2026_48979_MOESM1_ESM.pdf]

Qualisys PAF package: Functional Assessment marker set - Upper body

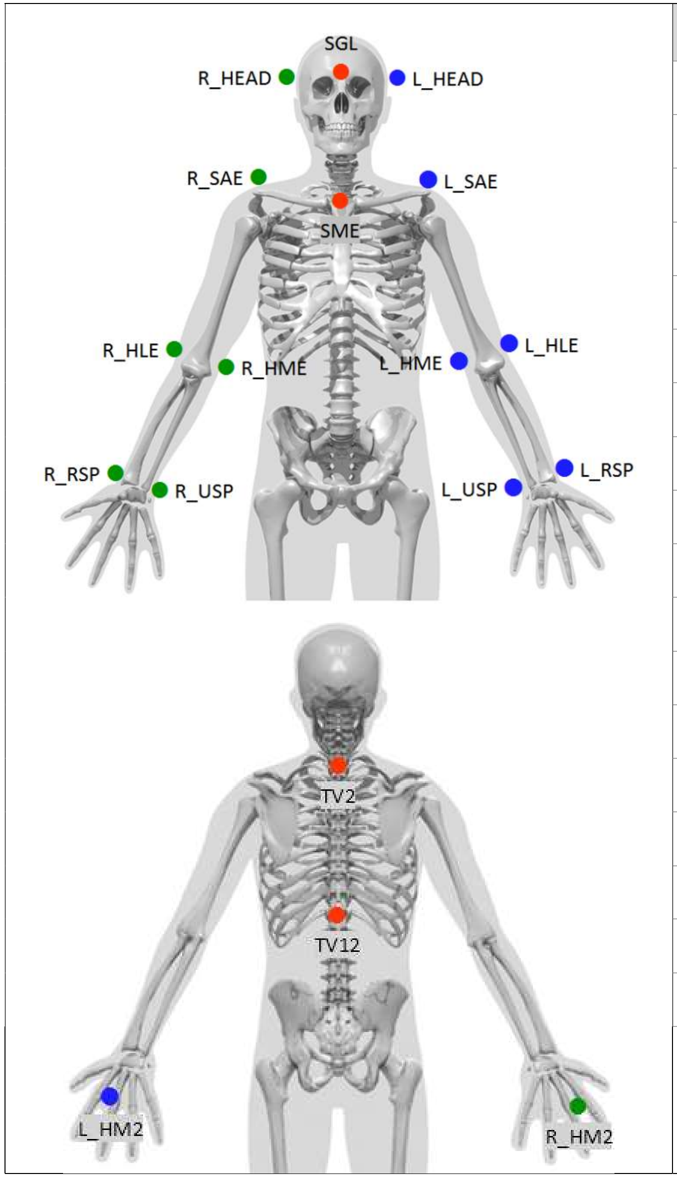

Name	Ref. [1]	Location	Static (18)	Dyn. (18)
L_HEAD		On headband, just above ear	X	X
R_HEAD		On headband, just above ear	X	X
SGL	SGL	On headband, Forehead	X	X
SME	SME	Sternum	X	X
TV2	TV2	Spine, 2nd Thoracic Vertebra	X	X
TV12	TV12	Spine, 12th Thoracic Vertebra	X	X
L_SAE	SAE	Shoulder	X	X
L_HLE	HLE	Elbow (outside)	X	X
L_HME	HME	Elbow (inside)	X	X
L_RSP	RSP	Wrist (thumb side)	X	X
L_USP	USP	Wrist (pinkie side)	X	X
L_HM2	HM2	Hand (basis of Forefinger)	X	X
R_SAE	SAE	Shoulder	X	X
R_HLE	HLE	Elbow (outside)	X	X
R_HME	HME	Elbow (inside)	X	X
R_RSP	RSP	Wrist (thumb side)	X	X
R_USP	USP	Wrist (pinkie side)	X	X
R_HM2	HM2	Hand (basis of Forefinger)	X	X

[1] Sint Jan, S. Van (2007). Color Atlas of Skeletal Landmark Definitions. Guidelines for Reproducible Manual and Virtual Palpations. Edinburgh: Churchill Livingstone.

Copyright © Qualisys AB

Qualisys PAF package: Functional Assessment marker set - Lower body

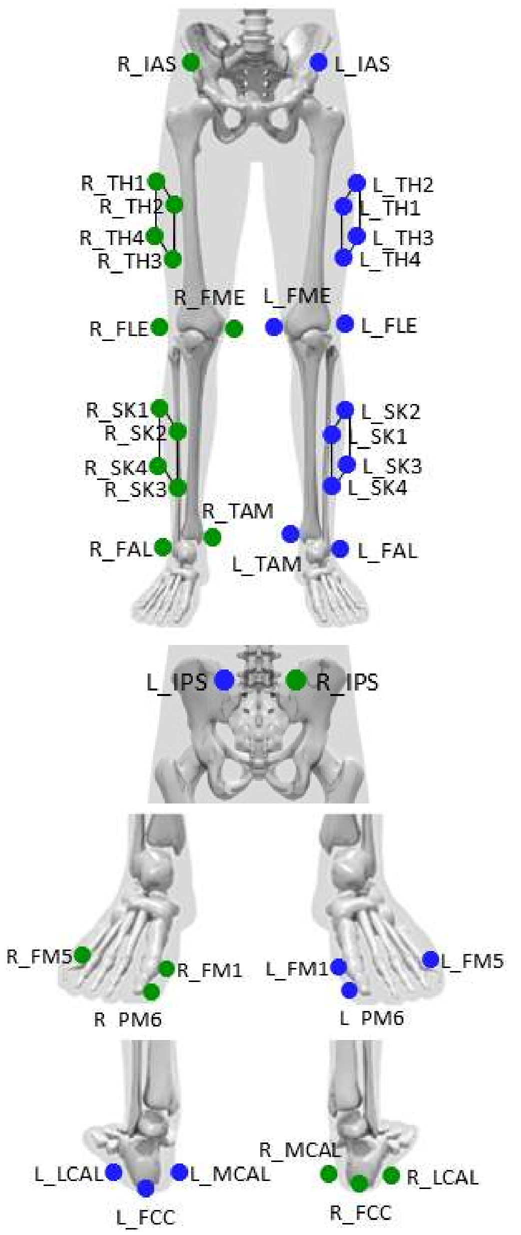

Name	Ref. [1]	Location	Static (28)	Dyn. (28)
L_IAS	IAS	Anterior superior iliac spine	X	X
L_IPS	IPS	Posterior superior iliac spine	X	X
R_IPS	IPS	Posterior superior iliac spine	X	X
R_IAS	IAS	Right anterior superior iliac spine	X	X
L_TH1-4		Cluster	X	X
L_FLE	FLE	Lateral epicondyle	X	X
L_FME	FME	Medial epicondyle	X	X
L_SK1-4		Cluster	X	X
L_FAL	FAL	Lateral prominence of the lateral malleolus	X	X
L_TAM	TAM	Medial prominence of the medial malleolus	X	X
L_FCC	FCC	Aspect of the Achilles tendon insertion on the calcaneus	X	X
L_LCAL		Lateral calcaneus	X	X
L_FM5	FM5	Dorsal margin of the fifth metatarsal head	X	X
L_PM6	PM6	Proximal medial phalanx of the big toe	X	X
L_FM1	FM1	Dorsal margin of the first metatarsal head	X	X
L_MCAL		Medial calcaneus	X	X
R_TH1-4		Cluster	X	X
R_FLE	FLE	Lateral epicondyle	X	X
R_FME	FME	Medial epicondyle	X	X
R_SK1-4		Cluster	X	X
R_FAL	FAL	Lateral prominence of the lateral malleolus	X	X
R_TAM	TAM	Medial prominence of the medial malleolus	X	X
R_FCC	FCC	Aspect of the Achilles tendon insertion on the calcaneus	X	X
R_LCAL		Lateral calcaneus	X	X
R_FM5	FM5	Dorsal margin of the fifth metatarsal head	X	X
R_PM6	PM6	Dorsal aspect of the second metatarsal head	X	X
R_FM1	FM1	Dorsal margin of the first metatarsal head	X	X
R_MCAL		Medial calcaneus	X	X

[1] Sint Jan, S. Van (2007). Color Atlas of Skeletal Landmark Definitions. Guidelines for Reproducible Manual and Virtual Palpations. Edinburgh: Churchill Livingstone.

Copyright © Qualisys AB

The final marker points application scheme adopted in the research

	L_HEAD		
	R_HEAD		
	SGL		L_IAS
	CV7		L_IPS
	L_SIA		L_IS
	R_SIA		R_IAS
	JN		R_IPS
	TV10		R_IS
	L_SAE		L_TH
	L_HUM1		L_FLE
	L_HUM2		L_FME
	L_HUM3		R_TH
Upper body	L_HLE	lower body	R_FLE
	L_HME		R_FME
	L_RSP		L_SK
	L_USP		L_FAL
	L_HM2		L_TAM
	R_SAE		R_SK
	R_HUM1		R_FAL
	R_HUM2		R_TAM
	R_HUM3		L_FT
	R_HLE		L_FCC
	R_HME		R_FT
	R_RSP		R_FCC
	R_USP		
	R_HM2		
